# LINC00978 predicts poor prognosis in breast cancer patients

**DOI:** 10.1038/srep37936

**Published:** 2016-11-29

**Authors:** Lin-lin Deng, Ya-yun Chi, Lei Liu, Nai-si Huang, Lin Wang, Jiong Wu

**Affiliations:** 1 Department of Breast Surgery, Fudan University Shanghai Cancer Center, 200032, China; 2 Department of General Surgery, the Second Affiliated Hospital of Nanchang University, Nanchang, 330006, China; 3 Department of Oncology, Fudan University, Shanghai Medical College, Shanghai, 200032, China; 4 Collaborative Innovation Center for Cancer Medicine, China

## Abstract

Breast cancer is the most frequently diagnosed cancer and the leading cause of cancer death among women worldwide. Long non-coding RNAs (lncRNAs) are a class of non-coding RNAs in the human genome that involves in breast cancer development and progression. Here, we identify a lncRNA, LINC00978, which is upregulated in breast cancer cell lines and tissues compared with corresponding controls. Furthermore, LINC00978 expression is negatively associated with hormone receptor (HR) status in 195 breast cancer patients studied (*p* = 0.033). Kaplan–Meier survival analysis shows that patients with high LINC00978 expression have poorer disease-free survival (DFS) than those with low LINC00978 expression (*p* = 0.012), and multivariate analysis identifies LINC00978 as an independent prognostic factor in breast cancer (*p* = 0.008, hazard ratio [HR] = 2.270, 95% confidence interval [CI] 1.237–4.165). Our study indicates that LINC00978 may be an oncogene in breast cancer, and can serve as a potential biomarker to predict prognosis in breast cancer patients.

Long non-coding RNAs (lncRNAs) are a class of non-coding transcripts more than 200 nucleotides in length^1^,^2^. Generally, lncRNAs can be divided into five categories: sense, antisense, bidirectional, intronic, and intergenic^1^,^3^. Increasing evidence shows that lncRNAs play critical roles in a wide range of cancers, including gastric cancer^4^, lung cancer^5^,^6^, glioma^7^,^8^, hepatocellular carcinoma^9^,^10^, and breast cancer^11^,^12^,^13^. LncRNAs can participate in chromatin remodeling, transcriptional controlling, post-transcriptional processing, protein function and localization, intercellular signaling in cancer^14^,^15^,^16^ as well. A growing body of evidence suggests that lncRNAs have a variety of functions, and further investigation is needed to a better understanding of their diverse roles.

Many lncRNAs have been shown to take important roles in tumorigenesis and can be used as biomarkers for cancers. For example, lncRNA HOX transcriptantisense RNA (HOTAIR) functions as an oncogene and an independent biomarker for diagnosis and prognosis prediction in various tumors including hepatocellular carcinoma^17^,^18^, lung cancer^19^,^20^, gastric cancer^21^,^22^, and colorectal cancer^23^. LncRNA metastasis-associated in lung adenocarcinoma transcript 1 (MALAT1) can also be used as a prognostic factor for survival and metastasis in various cancers^7^,^24^,^25^,^26^. H19 is aberrantly regulated in a great deal of cancers, it can serve as a biomarker for poor prognosis in patients with several tumor types^27^,^28^ as well.

An increasing number of lncRNAs have been found to have connections with breast cancer. Interactions between lncRNAs and drug treatment have also been drawing increased attention. Recent research demonstrates that BC200 can be induced by the estrogen receptor (ER) and recruit hnRNPA2/B1 to Bcl-x pre-mRNA to form a complex. The complex promotes the alternative splicing of Bcl-x to produce Bcl-xL, an anti-apoptotic factor. BC200 plays an oncogenic part in ER-positive breast cancer, while suppression of ER signaling^29^ with tamoxifen can decrease BC200 expression. Wu and colleagues find that lncRNA growth arrest-specific transcript 5 (GAS5), a tumor suppressor, acts as a microRNA (miRNA) sponge for miR-21 to upregulate expression of phosphatase and tensin homologs (PTEN), a tumor suppressor. Further study declares that GAS5 is downregulated in SKBR-3/Tr cells and breast cancer tissue from trastuzumab-treated patients, while lapatinib can upregulate GAS5 targeting of PTEN to relieve trastuzumab resistence^30^. Taken together, these researches suggest that lncRNAs have potential for acting as biomarkers in the clinical management of cancers.

LINC00978 (also named AK001796, MIR4435-2HG), located in the 2q13 region, is an oncogene and associated with chemoprevention medicine in lung cancer^31^. We searched several databases to collect comprehensive information of LINC00978. RNA Sequencing expression profile shows that Fragments Per Kilobase Million (FPKM) of LINC00978 in breast cancer samples is 1.027 (see Supplementary Fig. S1). Moreover, LINC00978 is related to ER and therapy from the Atlas of Noncoding RNAs in Cancer (TANRIC) integrative resource. This information indicates that LINC00978 plays an important role in breast cancer and is worth more attention. In this study, we aim to investigate the expression of LINC00978 in breast cancer cell lines and tissues, and to evaluate its potential as a prognostic biomarker in breast cancer patients.

## Results

### Expression spectrum of LINC00978 in breast cancer cell lines

**Figure 1:**
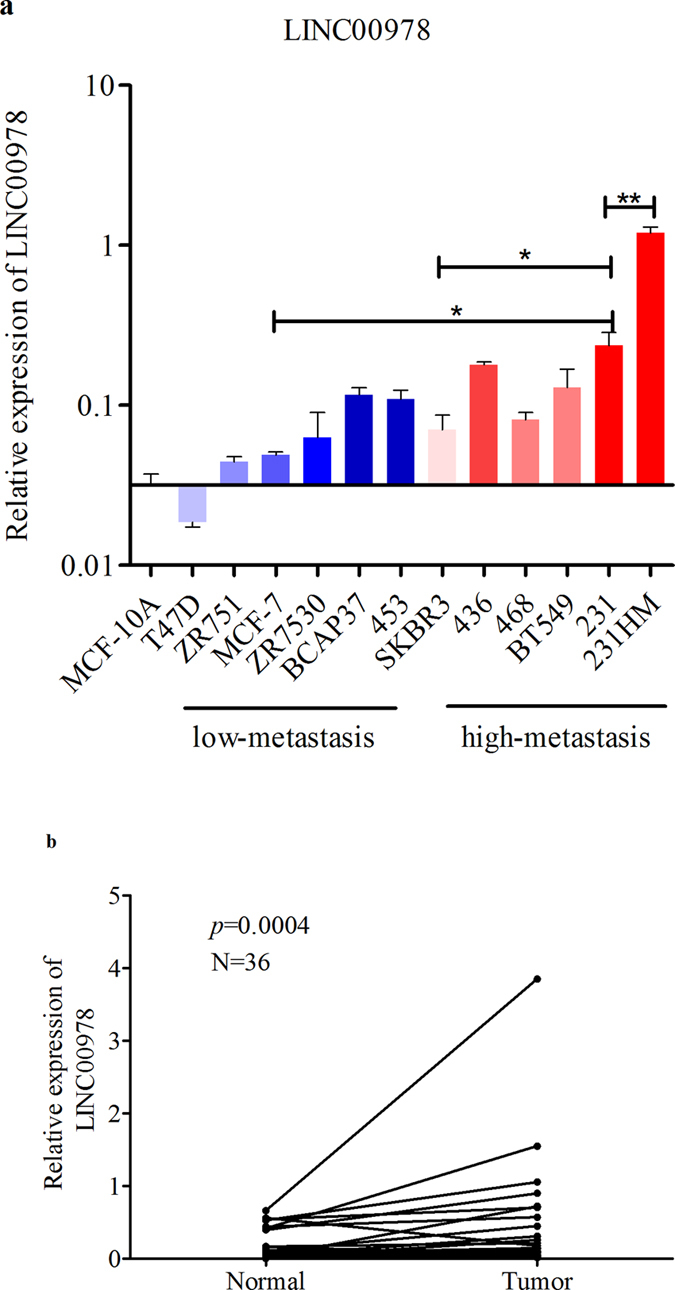
LINC00978 is highly expressed in breast cancer cell lines and tissues. (**a**) Comparison of LINC00978 expression between 12 cancer cell lines and the normal cell line 10 A by qRT-PCR. GAPDH was used as an internal control. (**b**) Comparison of LINC00978 expression in 36 pairs of breast cancer tissues and adjacent tissues. Abbreviations: 453, MDA-MB-453; 436, MDA-MB-436; 468, MDA-MB-468; 231, MDA-MB-231; 231HM, highly metastatic lung cancer cell line MDA-MB-231. *p < 0.05 **p < 0.01.

We first examined the LINC00978 expression profile in 12 breast cancer cell lines (Fig. 1a). Compared with the normal breast cell line MCF10A, LINC00978 was highly expressed in most cancer cell lines (11/12, 91.66%), including MCF7, BCAP37, SKBR3 and MDA-MB-231. We then divided these cell lines into low-metastatic and high-metastatic groups^12^,^32^. The low-metastatic group comprised T47D, ZR751, MCF-7, MDA-MB-453, BCAP37, and ZR7530, while the high-metastatic group included MDA-MB-436, SKBR3, MDA-MB-468, MDA-MB-231, MDA-MB-231HM (highly metastatic lung cancer cell line) and BT549. LINC00978 expression levels were significantly higher in the high-metastatic cell lines than in the low-metastasis cell lines (*p* = 0.026). LINC00978 levels were significantly higher in MDA-MB-231HM than in its parental cancer cell line, MDA-MB-231 (*p* = 0.001). These data suggest that LINC00978 may act as an oncogene and promote metastasis in breast cancer.

### LINC00978 is highly expressed in breast cancer tissues

We next examined LINC00978 expression in 36 pairs of breast cancer tissues and non-cancerous adjacent tissues. GAPDH was used as an internal control. 2^−ΔCt^ values were used to determine relative expression. LINC00978 showed significantly higher expression in cancer tissues than in normal tissues (*p* = 0.0004). These data further suggest that LINC00978 is prominently overexpressed in breast cancer, and that LINC00978 may facilitate breast carcinogenesis. (Fig. 1b).

### Correlations between LINC00978 expression and clinical characteristics in breast cancer patients

To identify the clinical relevance of LINC00978 expression in breast cancer, we examined the correlation between LINC00978 expression and clinicopathological characteristics in breast cancer tissues (Table 1). All of the patients studied have invasive ductal carcinoma. Expression levels of LINC00978 in tumor tissues were categorized as low or high using a cut-off value of 75%. Of the 195 breast cancer patients included in the study, 146 were categorized as low LINC00978 expression, and 49 were as high LINC00978 expression. LINC00978 expression in breast cancer was significantly correlated with hormone receptor (HR) status (*p* = 0.033). However, there was no obvious correlation between LINC00978 expression level and other clinical characteristics, such as TNM stage (*p* = 0.145), age (*p* = 0.240), or tumor size (*p* = 1.000).

### Role of LINC00978 in breast cancer patient survival

**Figure 2:**
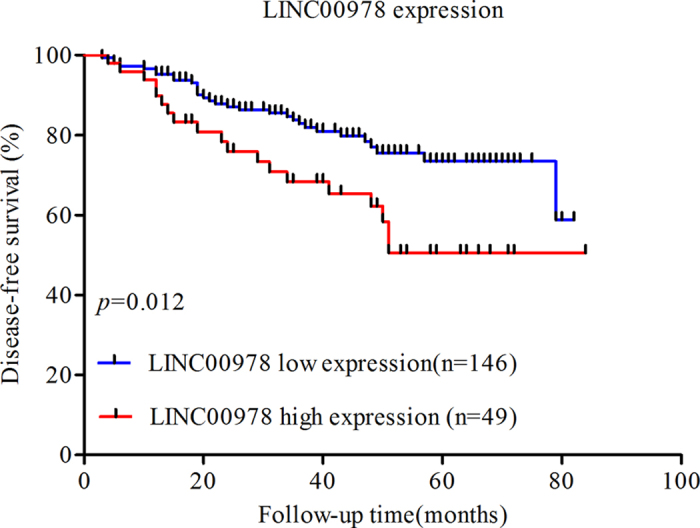
Kaplan–Meier survival curves of patients with breast cancer categorized by LINC00978 expression status.

In the 195 breast cancer patients included in the study, the median follow-up time is 47.6 months. Breast cancer recurred in 50 patients (25.64%), and 12 patients (6.12%) died (all as a result of breast cancer). Disease-free survival (DFS) was significantly poorer in patients with high LINC00978 expression than those with low LINC00978 expression (*p* = 0.012) (Fig. 2). Overall survival (OS) did not differ significantly between the two groups (data not shown).

We next performed univariate analysis of prognostic factors for DFS with the Cox regression model (Table 2). High LINC00978 levels were associated with worse DFS (*p* = 0.014, hazard ratio [HR] = 2.052, 95% confidence interval [CI] 1.158–3.636), larger tumor size (*p* = 0.030, HR = 2.103, 95%CI 1.073–4.120), and older patients (*p* = 0.008, HR = 0.462, 95%CI 0.262–0.815). Lymph node-positive disease was also associated with worse DFS (*p* < 0.001, HR = 3.373, 95%CI 1.725–6.595). We then performed multivariate analysis with Cox regression model (Table 3). LINC00978 expression (*p* = 0.008, HR = 2.270, 95%CI 1.237–4.165)), lymph node status (*p* = 0.002, HR = 3.037, 95%CI 1.503–6.136), age (p = 0.028, HR = 0.340, 95%CI 0.130–0.890) and human epidermal growth factor receptor-2(HER-2) status (*p* = 0.036, HR = 0.454, 95%CI 0.217–0.949) were independent prognostic factors in multivariate analysis. All of these data demonstrate that LINC00978 is a prognostic factor and high LINC00978 expression is associated with poor DFS.

## Discussion

Accumulating evidence shows that lncRNAs participate in cancer development and progression. Furthermore, ectopic lncRNA expression is associated with metastasis, invasion, and patient survival. However, a large number of unannotated lncRNAs still remain to be clarified, which requires further investigation of lncRNAs.

LINC00978, a long intergenic non-coding RNA, is located on chromosome 2q13. To date, its role in breast cancer has not been reported. Here, we found that LINC00978 was upregulated in breast cancer cells and tissues and its high expression in breast cancer patients was related to poor prognosis.

ER is a hormonal transcription factor that plays a vital part in breast cancer. Recently, loads of studies revealed the relationship among lncRNAs, ER and breast cancer. For instance, research has indicated that the estrogen–ERα–H19 signaling axis can regulate the proliferation and differentiation potential of normal luminal progenitor cells. Previous studies have shown that H19 was an oncogene in breast cancer. Thus, this signaling axis may play an important role in the development of ER+ breast cancer^33^. Singh and colleagues found that signaling via the ER can induce BC200 expression and thereby promote breast carcinoma development^29^. Researchers found that LINC00978 knockdown would cause a cell-cycle arrest in lung cancer^31^. They then examined the changes in gene expression by LINC00978 knockdown. Result shows that the cell-cycle-associated genes up- or down-regulated by LINC00978. In this study, we find that that LINC00978 expression level is significantly associated with HR status. What’s more, data on TANRIC shows that LINC00978 is significantly highly expressed in ER negative breast cancer (*p* = 0.00037) (see Supplementary Fig. S2). Hence, we speculate that LINC00978 may function through HR-related pathway to up- or down-regulated cell-cycle gene expression to promote breast cancer cell growth and proliferation. We will further investigate whether there are any other signaling pathways that mediate the association between LINC00978 and breast cancer in future studies.

Increasing attention has been attracted to the relationship between lncRNAs and drug resistance, for the development of chemotherapy resistance has become an obstacle in cancer treatment. The lncRNA HOTAIR is overexpressed in tamoxifen-resistant breast cancer compared to their primary counterparts, and is associated with poor clinical outcomes. ER is still recruited to chromatin in tamoxifen-resistant breast cancer, further binds to the HOTAIR promoter region and enhances transcription of the HOTAIR gene, thus promoting breast cancer growth. HOTAIR knockdown can significantly decrease tamoxifen-resistant cell growth. Therefore HOTAIR depletion may partially reverse tamoxifen resistance^34^. Similarly, Shang and colleagues demonstrate that silencing lncRNA UCA1 inhibit proliferation and resistance to adriamycin chemotherapy in gastric cancer^35^. Resveratrol (3,5,4-trihydroxy-transstilbene), has been shown to possess the ability of antiproliferative and proapoptotic effects in human cancer cells^36^. Resveratrol acts as an aromatase inhibitor, which makes it potential candidate in hormonal treatment of breast cancer^37^. Study shows LINC00978 may mediate resveratrol to inhibit the proliferation and growth of lung cancer^31^. Is there any relationship among LINC00978, aromatase inhibitor, and HR? Can we find a new path for breast cancer therapy? In addition to investigating the mechanisms by which LINC00978 is involved in breast cancer, we also intend to evaluate LINC00978 as a potential target for reduction of chemoresistance.

It’s a long way from applying LINC00978 to clinical work. Nowadays, many computational models have been developed to predict disease-lncRNA associations, which have shown effective prediction ability. For example, Fuzzy Measure-based LNCRNA functional SIMilarity calculation model (FMLNCSIM)^38^ and Improved LNCRNA functional SIMilarity calculation model (ILNCSIM)^39^ can obtain new lncRNA–disease association prediction. Gao *et al*. developed CSS sets to apply gene expression signature-defined high-risk stage II colorectal cancer (CRC) to predict survival^40^. These computational models will be involved in our future studies. With the development of multi-central big data, we will also pay attention to clinical data analysis and gene function annotation of LINC00978. It will get more accurate and robust predicting prognosis in LINC00978 relevant mechanism and clinical treatment study.

Our clinical data show that LINC00978 is upregulated in breast cancer cell lines and tissues. LINC00978 expression level is negatively associated with HR status. High expression of LINC00978 is positively associated with poor DFS. Our study indicates that LINC00978 may be an oncogene in breast cancer and has potential as a prognostic biomarker in breast cancer patients.

## Methods

### Patient’ samples

Two cohorts of patients were included. First, we obtained 36 pairs of primary non-metastatic breast cancer tissues and matched adjacent normal tissues. Second, data from 195 primary breast cancer cases treated at the Department of Breast Surgery in Fudan University Shanghai Cancer Center between 2008 and 2012 were collected. The exclusion criteria were: (i) advanced breast cancer, (ii) neo-adjuvant chemotherapy received prior to surgery, (iii) recurrent breast cancer diagnosed upon surgery, and (iv) noninvasive ductal carcinoma. Tissue samples were frozen in liquid nitrogen immediately after surgery and stored at −80 °C. Pathology, including histological type and grade; ER, PR, and HER2 status; and Ki-67 expression were assessed by two academic pathologists according to the WHO classification and ASCO guidelines. Basic patient information and details of tumor treatment were collected. All of the patients provided signed informed consent. The study was approved by the Ethical Committee of Fudan University Shanghai Cancer Center for Clinical Research (NO. 050432-4-1212B). We confirm that all methods were performed in accordance with the relevant guidelines and regulations.

### Cell culture

Twelve breast cell lines and one normal breast cell line (MCF10A) were purchased from ATCC. MCF10A cells were cultured in F12/DMEM 1:1 medium (HyClone, USA). MDA-MB-231 and MDA-MB-231HM cells were cultured in F15 medium (HyClone, USA). ZR751, ZR7530, MCF-7, SKBR3, BCAP37 and T47D cells were cultured in RMPI1640 medium (HyClone, USA). MDA-MB-436, MDA-MB-468, MDA-MB-453 and BT549 cells were cultured in DMEM medium (HyClone, USA). All cells were cultured with 10% fetal bovine serum (FBS) (Gibco, USA), 100 units/ml penicillin (Life technology, USA), and 100 ug/ml streptomycin (Life technology, USA) at 37 °C and 5% CO_2_.

### RNA isolation and quantitative RT-PCR

Total RNA was extracted from tissues and cell lines using Direct-zol™ RNA kits (Zymo research, USA) following the manufacturer’s protocol. The RNA concentration was examined using a NanoDrop 2000 spectrophotometer (Thermo scientific, USA). We then converted total RNA to cDNA in a reverse transcription (RT) reaction using PrimeScript™ RT Reagent Kit (Takara, Japan). To evaluate LINC00978 mRNA expression levels, we used quantitative real time polymerase chain reaction (qRT-PCR) with 2× TaqMan Premix Ex Taq (Takara, Japan), using an ABI7900 system (Applied Biosystems, USA). Dissociation curve analysis was evaluated the PCR products. GAPDH was used as an internal control, and 2^−ΔCt^ values were used to assess the relative expression of the target gene. The primers of GAPDH and LINC00978 were synthesized by Sangon Biotech (Shanghai, China).

qRT-PCR primers for LINC00978 were as follows:

Reverse primer (5′ to 3′): AGGCCCCAGGGAATCTTTCA

Forward primer (5′ to 3′): GCCTCTCCCTGAATAACTGGG

### Statistical analysis

Data were initially analyzed using Graph Pad Prism 5 (Graphpad Software Company, USA). The Mann–Whitney U test was used to analyze differences among groups. An unpaired t-test was used to analyze the difference between the MDA-MB-231 and MDA-MB-231HM cell lines. The Wilcoxon matched pairs test was used to compare LINC00978 expression in breast cancer tissues and matched non-cancerous tissues. We then used SPSS 17.0 software (SPSS Inc., Chicago, IL, USA) to further examine survival and correlation. Pearson’s *χ*^*2*^ test was performed to evaluate the correlation between breast cancer clinicopathological characteristics and LINC00978 expression. We used Kaplan–Meier analysis and the Cox proportional hazards model to examine whether LINC00978 expression levels were associated with prognosis in survival analysis. Two-tailed *p*-values were calculated, and *p* < 0.05 was considered statistically significant. We used Graph Pad Prism 5 (Graphpad Software Company, USA) to draw disease-free survival curve.

## Additional Information

**How to cite this article**: Deng, L.-l. *et al*. LINC00978 predicts poor prognosis in breast cancer patients. *Sci. Rep.* **6**, 37936; doi: 10.1038/srep37936 (2016).

**Publisher's note:** Springer Nature remains neutral with regard to jurisdictional claims in published maps and institutional affiliations.

## Supplementary Material

Supplementary Information

## Figures and Tables

**Table 1:** Relationship between LINC00978 expression and clinicopathological features in patients with breast cancer.

Characteristics	LINC00978 expression		N	X^2^	*p* value
	low	high			
Age(years)					
<50	56 (38.4%)	24 (49.0%)	80	1.711	0.240
≧50	90 (61.6%)	25 (51.0%)	115		
Menopause					
pre	62 (42.5%)	28 (57.1%)	90	3.180	0.097
post	84 (57.5%)	21 (42.9%)	105		
Tumor size*(cm)					
≦2.0	49 (33.8%)	16 (32.7%)	65	0.021	1.000
>2.0	96 (66.2%)	33 (67.3%)	129		
Node status					
negative	69 (47.3%)	21 (42.9%)	90	0.286	0.623
positive	77 (52.7%)	28 (57.1%)	105		
TNM stage					
I-II	108 (74.5%)	31 (63.3%)	139	2.269	0.145
III	37 (25.5%)	18 (36.7%)	55		
HR status					
negative	80 (54.8%)	18 (36.7%)	98	4.786	**0.033**
positive	66 (45.2%)	31 (63.3%)	97		
HER-2 status					
negative	98 (67.1%)	30 (61.2%)	128	0.566	0.489
positive	48 (32.9%)	19 (16.8%)	67		

Abbreviations: HR status, hormone receptor status; HER-2 status, human epidermal growth factor receptor-2 status; HR, hazard ratio.

^*^Only the size of invasive tumor included.

**Table 2:** Univariate regression model of prognostic covariates in patients with breast cancer.

Variable	95%CI		HR	*p* value
	Lower	Upper		
LINC00978 (high/low)	1.158	3.636	2.052	**0.014**
Tumor size*(>2/≦2 cm)	1.073	4.120	2.103	**0.030**
HR status(positive/negative)	0.705	2.302	1.274	0.422
HER-2 status(positive/negative)	0.307	1.129	0.588	0.110
Age(≧50/<50 years)	0.262	0.815	0.462	**0.008**
Menopause(post/pre)	0.325	1.011	0.573	0.054
Node status(positive/negative)	1.725	6.595	3.373	**<0.001**

Abbreviations: HR status, hormone receptor status; HER-2 status, human epidermal growth factor receptor-2; HR, hazard ratio; CI, confidence interval. Symbol bold data mean p < 0.05.

^*^Only the size of invasive tumor included.

**Table 3:** Multivariate analysis of clinicopathological factors for DFS in LINC00978 patients.

Variable	95%CI		HR	*p* value
	Low	High		
LINC00978(high/low)	1.237	4.165	2.270	**0.008**
Tumor size*(>2/≦2 cm)	0.997	3.873	1.965	0.051
Node status(positive/negative)	1.503	6.136	3.037	**0.002**
Age (≧50/<50 years)	0.130	0.890	0.340	**0.028**
HR status(positive/negative)	0.322	1.302	0.648	0.223
HER-2 status(positive/negative)	0.217	0.949	0.454	**0.036**
Menopause(post/pre)	0.649	4.543	1.717	0.276

Abbreviations: HR status, hormone receptor status; HER-2 status, human epidermal growth factor receptor-2; HR, hazard ratio; CI, confidence interval. Symbol bold data mean p < 0.05.

^*^Only the size of invasive tumor included.
