# Supplementary material for: LINC00978 predicts poor prognosis in breast cancer patients
Source: Sci Rep. 2016 Nov 29;6:37936. doi: 10.1038/srep37936 (PMC5126584; doi:10.1038/srep37936)

LINC00978 predicts poor prognosis in breast cancer patients

Lin-lin Deng[1,2,+], Ya-yun Chi[1,3,+], Lei Liu[1,2], Nai-si Huang[1,3], Lin Wang[2,*], Jiong Wu[1,3,4,*]

[1]Department of Breast Surgery, Fudan University Shanghai Cancer Center, 200032, China

[2]Department of General Surgery, the Second Affiliated Hospital of Nanchang University, Nanchang, 330006, China

[3]Department of Oncology, Fudan University, Shanghai Medical College, Shanghai, 200032, China

[4]Collaborative Innovation Center for Cancer Medicine, China

[+]These authors contributed equally to this work

[*]Corresponding authors: Jiong Wu, e-mail: wujiong1122@vip.sina.com; Lin Wang, e-mail: w71021@163.com

Supplementary Information

Figure. S1: Figure legends: RNA Seq Expression profile of LINC00978 in different cancer tissues.

Data resource: NONCODE database (http://www.bioinfo.org/noncode/).

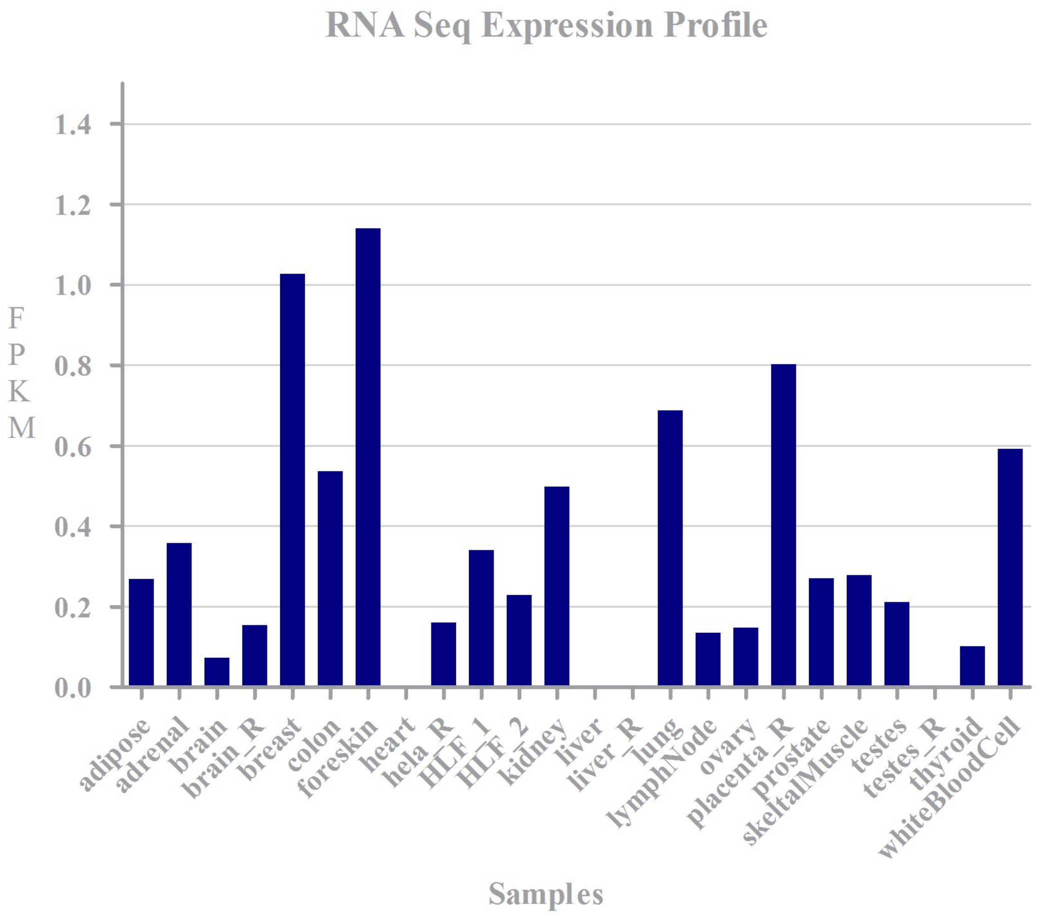

Figure. S2: lncRNA expression in different hormone receptor （HR）-status.

Data resource: TANRIC database

(http://ibl.mdanderson.org/tanric/_design/basic/analysis.html).

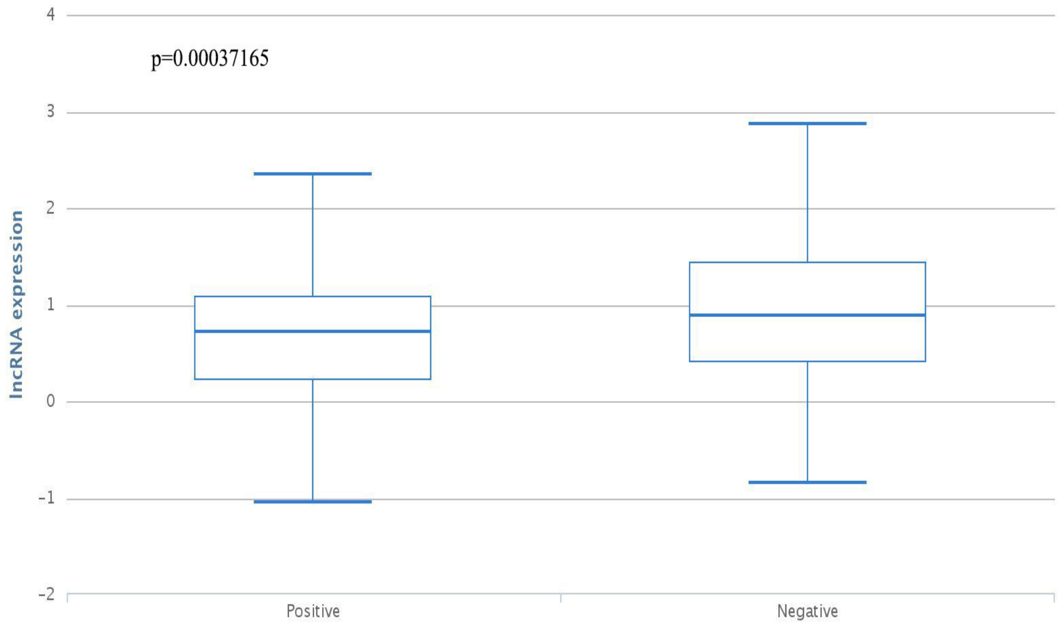

Supplement: Supplementary Information [file srep37936-s1.pdf]
